# Elucidation of complexity and prediction of interactions in microbial communities

**DOI:** 10.1111/1751-7915.12855

**Published:** 2017-09-19

**Authors:** Cristal Zuñiga, Livia Zaramela, Karsten Zengler

**Affiliations:** ^1^ Department of Pediatrics University of California, San Diego 9500 Gilman Drive La Jolla CA 92093‐0760 USA

## Abstract

Microorganisms engage in complex interactions with other members of the microbial community, higher organisms as well as their environment. However, determining the exact nature of these interactions can be challenging due to the large number of members in these communities and the manifold of interactions they can engage in. Various omic data, such as 16S rRNA gene sequencing, shotgun metagenomics, metatranscriptomics, metaproteomics and metabolomics, have been deployed to unravel the community structure, interactions and resulting community dynamics *in situ*. Interpretation of these multi‐omic data often requires advanced computational methods. Modelling approaches are powerful tools to integrate, contextualize and interpret experimental data, thus shedding light on the underlying processes shaping the microbiome. Here, we review current methods and approaches, both experimental and computational, to elucidate interactions in microbial communities and to predict their responses to perturbations.

## Introduction

Microorganisms form complex and diverse communities, which inhabit almost every environment on Earth. These microbial populations interact with each other in many different ways, creating complex and intertwined networks. The interaction between living cells and their environment forms the foundation for microbial diversity. In nature, microbial diversity is often accompanied by vast metabolic capability reflected in fundamental biogeochemical processes (McCalley and Sparks, 2009; Guidi *et al*., 2016; Louca *et al*., 2016). Microorganisms also interact directly with higher organisms. The recent boost in studies of host–microbe interactions has shed light on the crucial role the human microbiome plays in health and disease. The human microbiota shapes significant parts in the host physiology and provides essential functions, e.g. nutrient catabolism (Flint *et al*., 2012), syntheses of vitamins (Degnan *et al*., 2014) and modulation of the human immune system (Fischbach and Segre, 2016). Albeit the importance of microorganisms to human health and evolution, we currently possess limited knowledge about how microbes interact amongst each other and with their host and how these interactions contribute to health. Additionally, the response of microbial communities to perturbations (e.g. nutrient availability or response to environmental shifts) is poorly understood, thus limiting our ability to predict the responses of the microbiome to interventions.

**Figure 1:**
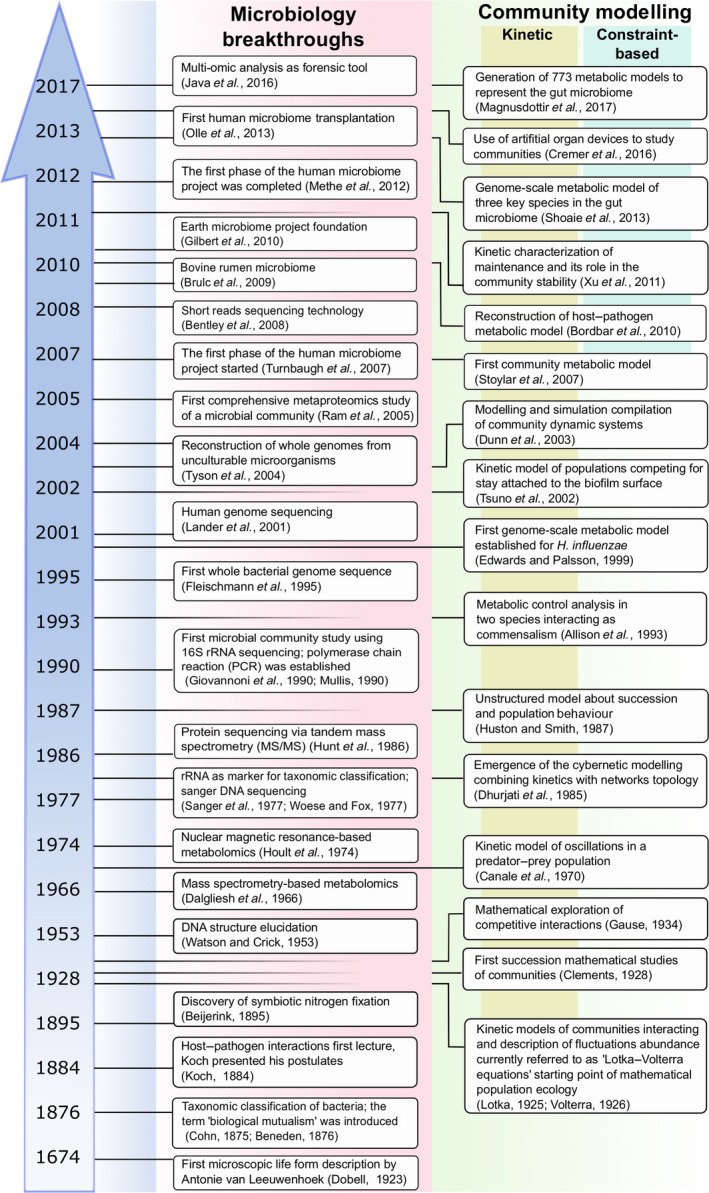
Milestones in microbiology and computational modelling for the study of microbial communities.

For centuries, most of our knowledge that paved the way for our understanding of microbial cells has been gathered through approaches that focused on single microorganisms, i.e. pure cultures (Fig. 1). Even with the introduction of advanced molecular biology techniques, most work has been performed on cultures in isolation, studying single molecules, such as single genes, transcripts, proteins, metabolites (Fleischmann *et al*., 1995; Lander *et al*., 2001). While pioneers in microbial ecology described complex microbial systems early on, using e.g. microscopy and enrichment cultures (Cohn, 1875; Winogradsky, 1887; Beijerinck, 1895; Dobell, 1923), they were limited by the technical methods available at the time. Only recently have we been able to apply molecular methods to study complex microbial systems *in situ*, thereby gaining insight into the complexity of microbial networks (Fig. 1). In sync with the development of new experimental methods has been the advancement in computational methods, which enables us to contextualize complex data types. Systems biology, the computational modelling of biological systems deploying a holistic approach, emerged as an interdisciplinary field that enables the study of interactions between various components of a living cell (Bordbar *et al*., 2014). While first applied to single organisms, systems biology approaches have been expanded to study the microbiome.

The comprehension of microbial communities in the context of systems biology beyond the description of individual parts of a living cell is a relatively new concept (Zengler and Palsson, 2012). In order to help unravel properties inherent to microbial interactions, systems biology approaches dynamically and simultaneously investigate the multiple operational components of the community. The emergence of culture‐independent omic approaches, e.g. metagenomics, metatranscriptomics, metaproteomics and metabolomics, has increased our ability to probe microbial community composition and function *in situ*. These multi‐omic approaches have changed our ability to investigate biological systems, providing essential experimental data to reconstruct and constrain computational models. At the same time, integrative data analysis methods, fundamental to elucidate mechanisms beyond individual interactions in microbial communities, have been developed and deployed (Embree *et al*., 2015). Lastly, studying the microbiome using systems biology tools, such as predictive computational models, allows generating testable hypothesis on a community level (Magnúsdóttir *et al*., 2016). While systems biology approaches have successfully been deployed for single organisms to design and optimize the production of diverse products such as antibiotics, alcohols and amino acids, these approaches can potentially be extended to multispecies communities (Perez‐Garcia *et al*., 2016; von Kamp and Klamt, 2017). In this review, we discuss the current state of fundamental mechanisms elucidating and shaping the diversity and structure of microbial communities. We will highlight relevant biotechnological examples applying model‐driven multi‐omic analysis. Furthermore, we will provide an outlook on the development of predictive models for complex communities combining the use of omic tools in an iterative design–build–test–learn cycle.

## Types of interactions

**Figure 2:**
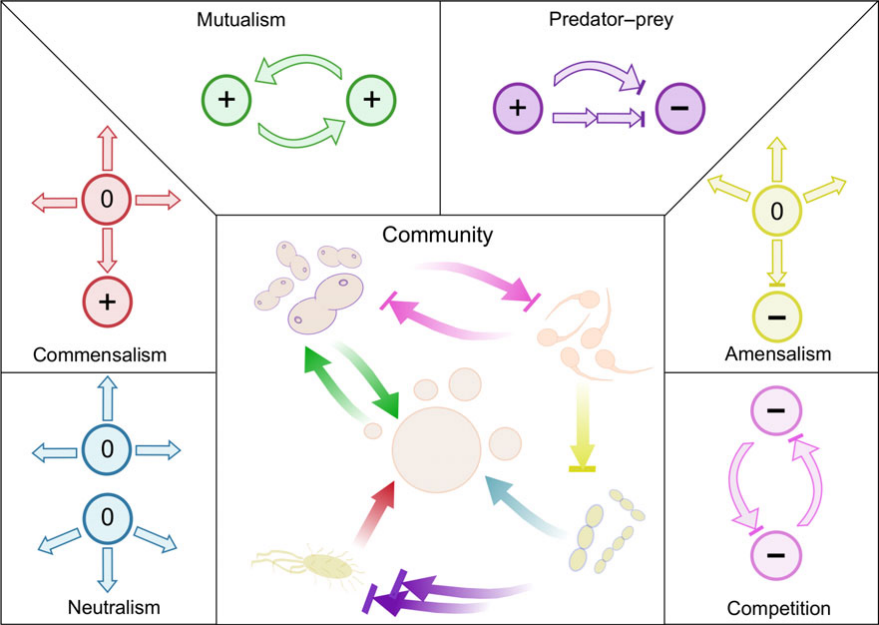
Interactions amongst microorganisms. Within a complex community, the members of the population may engage in multiple interactions at the same time. 0 refers to neutral interaction, while + and – depicts a positive or a negative outcome of interaction.

In nature, organisms rarely exist in isolation. Rather, they form complex relationships that are developed through evolution and adaptation to diverse ecosystems (Dunn *et al*., 2003; Tan *et al*., 2015). This complex network of interactions defines how a community is assembled and maintained spatially and temporally. Different types of interactions have been defined based on the benefit for an organism. Interactions thus can be beneficial, neutral or disadvantageous for the individual organism (Fig. 2). The best‐studied type of interaction in microbiology is beneficial mutualism. A prime example for this type of interaction is syntrophy (Morris *et al*., 2013), in which all community members are sustained by the activity of the other(s). One example of mutualism is the host–bacterial interaction in the human intestine, where the host gains energy from short‐chain fatty acids released by the microbes through fermentation of glycans provided by the host (Backhed *et al*., 2005). Another interaction type, in which one member benefits and the other neither benefits nor has a disadvantage, is known as commensalism. Examples of commensalism include, for example, nitrification and methanogenesis (Allison *et al*., 1993). Commensalism and mutualism can also include interactions where one organism consumes specific metabolites that are inhibitory for another organism (Dunn *et al*., 2003). Cross‐feeding of inhibitory metabolites is a variation of mutualism and commensalism where the products of one organism are the substrate for another and the uptakes of those become beneficial to one member (Germerodt *et al*., 2016). Amensalism on the other hand is a relationship in which one member is disadvantaged and the other neither benefits nor is harmed. In plant–microbe communities, amensalism is often referred to as allelopathy and can involve the natural secretion of secondary metabolites, which inhibits the growth of surrounding microorganisms (Chaparro *et al*., 2014). When members of a local environment do not significantly affect each other, the interaction is termed neutralism (Dunn *et al*., 2003). If one population member is disadvantaged and another benefits, the interaction is termed predator–prey or parasitism. This relationship has been extensively studied throughout all forms of life (i.e. eukaryotes including plants, bacteria and viruses). Apparently, predator–prey interactions can stabilize populations by creating a cyclic trend between species as well as preventing the depletion of resources by fast‐growing species (Canale, 1970; Alhumazi and Ajbar, 2005; Chen *et al*., 2011). Finally, the interaction type in which all members are disadvantaged by the presence of others is referred to as competition (Gause, 1934). A model case of competition is two populations of autotrophic and heterotrophic bacteria compete for the oxygen, thus promoting a spatial competition for the attachment to the surface (Tsuno *et al*., 2002). Defining these basic forms of interactions in complex communities can be a challenge as interactions are prone to change over time due to changing physiological and physical parameters such as nutrient availability. Furthermore, interactions can be dependent on the spatial organization of members of a community.

## Multi‐omic approaches to study microbial communities

**Figure 3:**
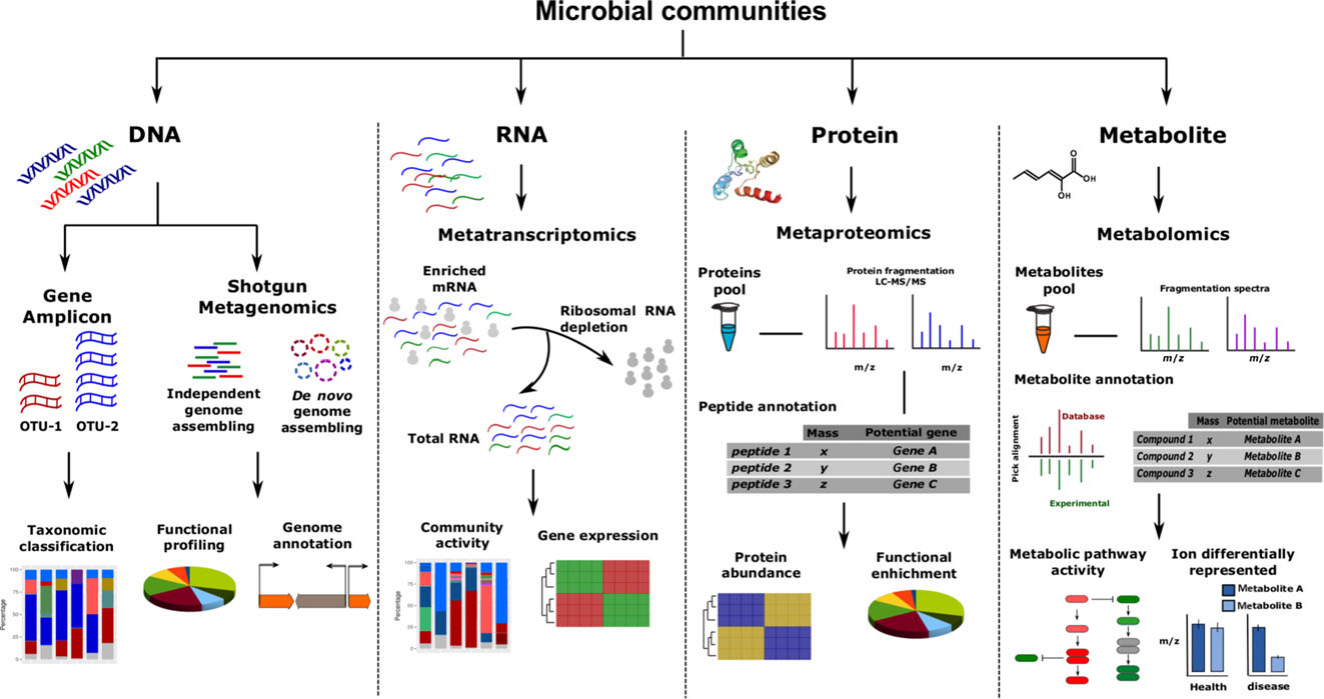
Overview of different omics techniques. Different experimental approaches and computational strategies are applied for the study of microbial communities. During gene amplicon analysis, SSUs or ITS sequences are clustered into operational taxonomic units (OTUs) and the taxonomic identity is assigned for each OTU based on sequence homology against known sequences in a database. The resulting OTUs are used to calculate the relative abundance of each organism and quantify the population diversity between samples. In shotgun metagenomics, genomic DNA sequences can either be mapped to a reference database or used for *de novo* assembly of genomes. The recovered genomes can be used to assign phylogeny, calculate the relative abundance of the identified genome and assess the functional capability. In metatranscriptomics, messenger RNA (mRNA) is used to generate complementary DNA libraries that can either be mapped to reference genomes to generate gene expression profiles. These expression profiles are used to identify active pathways, genes and organisms. In metaproteomics, mass spectrometry and fragmentation are used to reveal the amino acid sequence of peptides. The identified peptides are associated with full‐length proteins by sequence homology searches against a reference database. Similar to metatranscriptomic analysis, protein expression profiles can be used to identify active pathways as well as active organisms. In metabolomics, metabolites are separated using chromatography techniques and identified and quantified using mass spectrometry. Similar to metaproteomics, the comparison between fragmentation profiles and reference databases is used to annotate the metabolic compound. Enrichment and clustering analysis can be applied to reveal patterns between sets of samples or to identify condition‐dependent compounds.

Microbiome research has greatly benefited from recent breakthroughs in sequencing technologies as well as in improvements in proteomics and metabolomics, and accompanying data analysis tools (Franzosa *et al*., 2015; Jansson and Baker, 2016). Gene amplicon sequencing, metagenomics and metatranscriptomics are currently applied to elucidate microbial community composition and function and to determine potential interactions (Fig. 3). While these methods promoted recent advances in microbiome research, they can only provide limited mechanistic insight (by itself) into the determination of the mode of interaction. In combination with computational tools and modelling approaches, these experimental approaches can be used to unravel intertwined interactions, provide mechanistic knowledge and predict community behaviour. In this section, we will discuss advantages and disadvantages of different omic approaches for the study of microbial communities and their potential application for quantitative analysis by computational modelling.

## Gene amplicon sequencing

The first studies interrogating microbial community composition emerged decades ago, through the comparative analysis of ribosomal RNA (Lane *et al*., 1985; Giovannoni *et al*., 1990; Delong, 1992; Fuhrman *et al*., 1992; Fig. 1). Gene amplicon sequencing is currently the fastest assay to perform taxonomic identification and phylogenetic profiling of microbial communities. This technique consists of genomic DNA extraction, followed by amplification of a conserved genomic region and sequencing. Phylogenetic classification based on gene amplicon sequencing of small ribosomal subunits (SSUs) for bacteria, archaea and eukaryotes (16S or 18S rRNA gene) or internal transcribed spacer (ITS) region for fungi are routinely applied to describe operational taxonomic units (OTUs) in microbial ecology (Walters *et al*., 2016). SSUs are highly conserved sequences intercalated by hypervariable regions that can be used as phylogenetic marker. In addition, sequencing of hypervariable regions instead of the full‐length gene became very popular for profiling microbial communities (Yang *et al*., 2016). This technique has been successfully employed to investigate the taxonomic composition and phylogenetic diversity for very different samples, from geothermal springs (Ward *et al*., 2017) and oil reservoirs (Lewin *et al*., 2014; Sierra‐Garcia *et al*., 2016) to the human microbiome and host–microbiome‐related diseases (Boguniewicz and Leung, 2011; Halfvarson *et al*., 2017). The success and low cost of amplicon sequencing encouraged major initiatives, such as the Human Microbiome Project (HMP), American Gut Project (AGP) and the Earth Microbiome Project (EMP; Turnbaugh *et al*., 2007; Gilbert *et al*., 2010; McDonald *et al*., 2015). These large sequencing efforts have already generated taxonomic profiles for tens of thousands of samples from different sides of the human body, the human gastrointestinal tract, as well as diverse samples from around the world.

**Figure 4:**
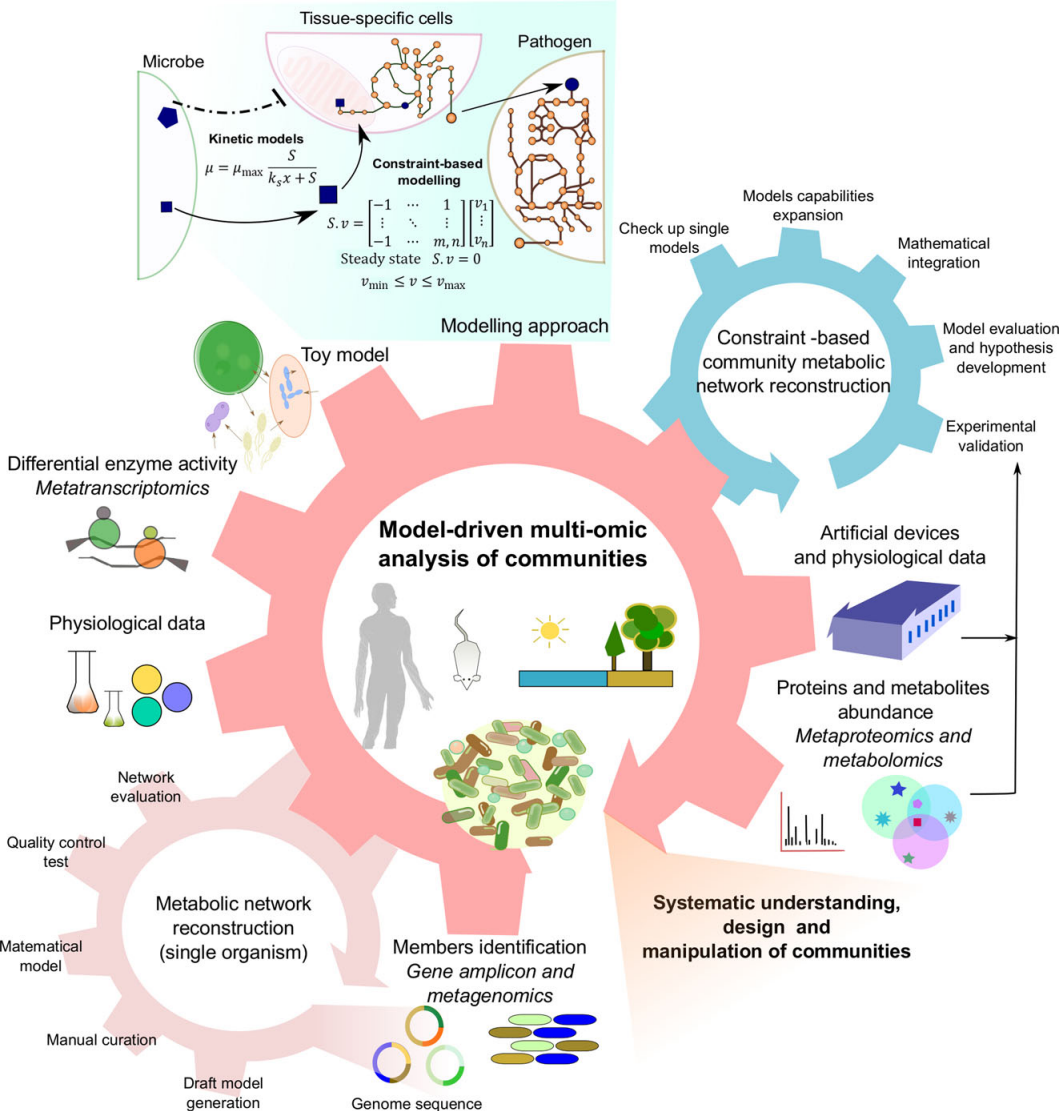
Integration of experimental data and *in silico* analysis in a community systems biology approach. The first step in the iterative workflow encompasses the identification of individual community members, followed by the creation and validation of single metabolic networks. Subsequently, a predictive community model is constructed and validated. Various experimental and computational methods are interspersed along the design–build–test–learn cycle to unravel community interactions and predict community dynamics.

The advantages of gene amplicon‐based sequencing are low cost, easy sample preparation protocols and broad availability of bioinformatics tools (Caporaso *et al*., 2010). Although the gene amplicon sequencing has provided unprecedented insight into microbiome composition, this technique can only provide community member profiling and indirect insights into functionality through comparison with reference genomes (Brooks *et al*., 2015). While universal primers can readily be designed to cover bacterial and archaeal 16S rRNA genes, 18S rRNA and ITS regions in eukaryotes are more variable than 16S rRNA genes in bacteria and archaea, which makes universal primer design and thus eukaryotic diversity surveys challenging (Wang *et al*., 2014). Additionally, shallow resolution based on amplicon size makes it difficult to identify closely related species belonging to the lowest level of the taxonomic classification or in some cases to the same genus. While unambiguous identification at the strain level is not possible by 16S rRNA (Brooks *et al*., 2015), this resolution is often critical for diagnostic in human health, e.g. to identify and discriminate a certain pathogen from a commensal strain. The importance of accurate identification at strain level has been highlighted in model‐driven analysis of multiple bacteria strains, such as *Staphylococcus aureus* (Bosi *et al*., 2016) and *Escherichia coli* (Monk *et al*., 2013). These studies found significant differences in metabolic capabilities amongst different strains, such as amino acids and vitamin autotrophies, which can be related to pathogenesis. The correct strain identification is vital for the systematic understanding of microbial communities, which is the first step in the iterative cycle to unravel community interactions and dynamics (see Fig. 4).

## Whole‐metagenomic shotgun sequencing

Whole‐metagenomic shotgun sequencing is a powerful metagenomics technique to study microbial communities beyond describing OTU composition of a sample. For this, genomic DNA from all cells in a community is extracted, sheared into short fragments and subsequently sequenced. The short DNA sequences (i.e. reads), usually smaller than 250 bp, can be used to taxonomic classification by recovering the reads aligned to taxonomically informative genomic loci (16S or 18S rRNA, ITS or conserved proteins; Meyer *et al*., 2008; Truong *et al*., 2015), to estimate relative microbial abundance, to generate functional profiling and to reveal genomic content of unknown microbes (Abubucker *et al*., 2012; Hug *et al*., 2016). The identification of unknown microorganisms usually depends on *de novo* genome assembling methods that combine overlapping reads to generate contiguous fragments of DNA sequence (i.e. contigs). Assembling methods can be time‐consuming, require robust computational resources and deep‐sequencing data sets, and rely on differential binning tools to group contigs into potential genomes (Jansson and Baker, 2016). Despite this, *de novo* genome assembling is fundamental to create compendium genomes representing not yet cultured microorganisms, to generate comprehensive organism‐specific metabolic mapping (Sangwan *et al*., 2016) and to detect larger complex genomic features such as polyketide synthase (PKS), cluster regulatory interspaced short palindromic repeats (CRISPR) and non‐ribosomal peptide synthase (NRPS) gene clusters (Vollmers *et al*., 2017).

Another, less time‐consuming analysis method for metagenomics data is the reference‐based mapping method, which facilitates comparisons amongst multiple samples (Abubucker *et al*., 2012). This method usually involves mapping reads to reference genomes and interpreting the mapping results in terms of relative abundance and functional profiles of the sample. Hence, independent genome assembly outcomes depend heavily on genome reference databases and often present high false‐positive rates due to regions of local homology (Carr and Borenstein, 2014). Both metagenomic analysis approaches have been applied to simultaneously investigate the metabolic requirement of individual members in a microbial community and to determine potential microbe–microbe and microbe–host interactions (Greenblum *et al*., 2012; Embree *et al*., 2015).

*De novo* genome assembling studies have been used for downstream community modelling (Biggs *et al*., 2015; Embree *et al*., 2015; Magnúsdóttir *et al*., 2016). The metagenomic results were used as a genome platform to reconstruct and validate genome‐scale metabolic networks for these communities. In turn, independent genome assembly methods have been extensively used to infer the metabolic repertoire of different microbiomes (Greenblum *et al*., 2012; Levy and Borenstein, 2013). Usually, experimental data is analysed using a metabolic analysis network, in which the microbiome is treated as a single ‘independent’ biological system (Abubucker *et al*., 2012; Greenblum *et al*., 2012). This approach does not take into account the contribution of each organism in the community, ignoring the boundaries between cells and compartmentalization of various metabolites. Although simplified models are subject to inaccuracies and noises, they can provide valuable insight into the metabolic potential of the entire system (Ji and Nielsen, 2015). For example, topological analysis of metabolic networks, observing changes in connections and centrality of the nodes (genes or enzymes), can be helpful to identify genes/functions associated with different conditions (Tan *et al*., 2015; Steffani‐Vallejo *et al*., 2017).

While metagenomic sequencing can provide relevant information related to the taxonomic profile and the metabolic capability of a community, the data do not provide information about viability of the microbial community members. It can be essential to complement sequencing with experimental methods that distinguish metabolically active from inactive cells. Several methods targeting active microbial cells have been discussed in recent reviews (Franzosa *et al*., 2015; Singer *et al*., 2017). *In situ* microbial activity methods can be potentially applied to study microbial population. These methods can differ dependent on the target molecule (DNA, RNA, protein and metabolites) and the biological process to be investigated (DNA replication, cell division, transcription, amino acid biosynthesis). Most of these techniques such as bromodeoxyuridine labelling, DNA‐SIP (stable isotope probing), iREP (index of replication), RNA‐SIP, protein‐SIP are usually restricted to samples with low complexity or provide information about a limited number of community members. Furthermore, the methods are generally associated with high cost and require immediate processing of the sample. In addition, applying these techniques to studies in human can be challenging (Singer *et al*., 2017).

## Metatranscriptome: transcriptional expression in microbial communities

Conventional transcriptomic techniques measure messenger RNA (mRNA) levels in a simple biological sample (usually a single organism exposed to different conditions) and analyse the activity, i.e. differential expression, under these scenarios (Fig. 3). Metatranscriptomic approaches are an extension of this method and are measuring mRNA levels for all microbes in a given community. Accessing the expression level of the microbiome allows for determining its functional profile, inferring the biological role, and thus helps to decipher the type of interactions microbes are engaged in and to relate this information to metabolic pathways. This technique has been successfully applied to study diverse microbial communities. For example, metatranscriptomics has been used to determine the relationship between the oral and the gut community (Franzosa *et al*., 2014) and to study human microbiome‐related diseases, e.g. periodontitis (Jorth *et al*., 2014), colorectal cancer (Dutilh *et al*., 2013) and acne vulgaris (Kang *et al*., 2015).

Transcriptomic data have been used extensity to constrain metabolic models of single cultures or co‐cultures and to gain insight into metabolic activity and predict metabolic fluxes (Bordbar *et al*., 2014; Zielinski *et al*., 2015). These model‐driven analyses provided a mechanistic interpretation for the better understanding of the relationships between diet, microbiota and host (Shoaie *et al*., 2013). Computational and mathematical approaches, as transcriptional networks, have emerged as potential alternative to elucidate microbe–microbe interaction in complex communities. These approaches were extended to investigate transcriptional interactions in the human gut microbiome (Plichta *et al*., 2016). Community‐wide transcriptional regulation is suggested to be a mechanism for reducing competition and for niche segregation (Zelezniak *et al*., 2015). For example, germ‐free mice colonized with two major bacteria of the human distal gut, *Eubacterium rectale* and *Bacteroides thetaiotaomicron*, were used to demonstrate how these bacteria change the whole‐transcriptional profile to adapt to the presence of each other (Mahowald *et al*., 2009). A similar approach was applied to analyse the metatranscriptomics data from 233 human faecal samples. Transcriptional interactions between pairs of coexisting gut microbes revealed the transcriptional changes, which led to a reduced expression of orthologous functions between interacting species pairs. Specific species–species transcriptional interactions were enriched for essential functions, such as butyrate biosynthesis, ATP‐binding cassette (ABC) transporters, flagella assembly and bacterial chemotaxis (Plichta *et al*., 2016).

Metatranscriptomic approaches are advantageous for the study of microbial communities *in situ*. Despite this, the approach has its pitfalls, one of them being the common practice to use mRNA expression levels as proxy for levels of corresponding proteins (Guimaraes *et al*., 2014). Comparisons amongst transcriptional data and proteomics data from different biological samples showed that the correlation between mRNA levels and protein abundance can vary dramatically (0.2–0.9; Maier *et al*., 2009). This nonlinear relationship can be explained by various reasons, such as half‐life time of RNAs and proteins, post‐transcriptional and translational efficiency and experimental error and noise. Ribosomal footprint profiling (RFP) approach that uses the ribosomal machinery genomic occupancy to provide a high‐resolution quantitative profile of translation across the transcriptome can circumvent part of these obstacles (Latif *et al*., 2015; Ingolia, 2016). However, up to now this method has not been applied to any microbiome study.

## Metaproteomics: large‐scale characterization of the protein content in microbial communities

Metaproteomics approaches aim to measure protein abundance in all cells in a microbial community. Usually, proteomic methods consist of three main steps: sample fractionation, protein separation and protein identification/quantification. Technological advances of molecular separation methods coupled with mass spectrometry enabled the study of the proteome of microbial communities. Such advances include two‐dimensional polyacrylamide gels coupled with mass spectrometry (2D nano‐LC/MS‐MS), liquid chromatography coupled with mass spectrometry (LC‐MS/MS) and matrix‐assisted laser desorption/ionization (MALDI) coupled with time‐of‐flight mass spectrometer (TOF). Metaproteomics has been applied to examine different microbial communities from various environments (Ng *et al*., 2010; Zampieri *et al*., 2016), including the human gut, small intestine, colon, liver and adipose tissue (Mardinoglu *et al*., 2015; Xiong *et al*., 2015).

For example, metaproteomics was applied to understand the symbiotic relationship between human cells and the microbial community in the gastrointestinal tract (Young *et al*., 2015). The proteome of human cells and microbes was simultaneously monitored over time throughout early development of the preterm infant microbiome. This study revealed that in response to early microbiome colonization, human cells synthesize an increased amount of proteins responsible for epithelial barrier function and antimicrobial activity. In addition, neutrophil‐derived proteins were detected in high abundance, indicating activation of the human innate immune response (Young *et al*., 2015). Similar to transcriptomic data, proteomic data have been successfully applied to constraint metabolic models and increase the prediction accuracy (O'Brien *et al*., 2014), as well as to reconstruct tissue‐specific models. Twenty‐eight metabolic models were reconstructed for the small intestine, colon, liver and adipose tissues based on proteomics data. These model‐driven proteomics analyses showed that the gut microbiota directly influences host amino acid and glutathione metabolism in mice (Mardinoglu *et al*., 2015).

Although recent technological advances in mass spectrometry have allowed high‐throughput proteome measurement in a fast and cost‐effective way, this technique has technical limitations. One of them is the protein identification resolution. For example, the estimated number of unique proteins in human‐associated microbiome samples is in the order of millions, but only a few thousand can be identified using current methods (Erickson *et al*., 2012). The assessment of proteins with different features, e.g. different solubility or trans‐membrane domain, requires specific protocols and multiple sample preparations to generate a complete proteome map. Various alternative methods to increase proteome coverage and resolution have become recently available (Wilmes *et al*., 2015; Vincent and Postovit, 2017).

## Metabolomics: tapping into the metabolic diversity

Metabolite exchange is often assumed to be a primary driver shaping microbial communities. While changes in transcript levels and protein abundances can usually be associated with one or a few genes, often changes in metabolic fluxes and metabolite levels are the final result of complex interactions (Palsson and Zengler, 2010). Metabolites produced and secreted by living cells have tremendous physicochemical diversity, and can usually differ by multiple orders of magnitude in concentration. Due to the dynamic nature of these compounds, their concentrations and compositions can change rapidly, which makes detection and data analysis a challenging task (Ponomarova and Patil, 2015). Hence, the detection of all metabolites in a biological sample requires a combination of several sample preparation methods and often analysis on multiple platforms. Although thousands of metabolites can be detected in a single experiment, <2% of all compounds from an untargeted metabolomics experiment can currently be annotated (Silva *et al*., 2014).

Improved tools to annotate and integrate metabolomics data sets have been recently developed. One of them is the GNPS (Global Natural Products Social Molecular Networking) that similar to BLAST (Basic Local Alignment Search Tool) for nucleotides and amino acid sequences, assists the storage, analysis and dissemination of public MS/MS data, hence contributing in the identification of known compounds (Wang *et al*., 2016). Another tool is XCMS online that provides simplified metabolomics data processing and analysis (Gowda *et al*., 2014) and more recently allows multi‐omics analysis by integrating metabolic pathway information with metabolomics, metatranscriptomics and metaproteomics data (Huan *et al*., 2017). Despite some of these limitations and others regarding technical and analytical requirements, metabolomics is the method of choice for the discovery of new antibiotics (Nothias *et al*., 2016) and to evaluate the effect of bacterial metabolites on human health (Wang *et al*., 2011). Host–microbe metabolomics is an emergent area in systems biology with a high potential impact on the discovery of novel drugs and biomarkers (Heinken and Thiele, 2015). The use of metabolomics has been fundamental in identifying new biomarkers for various human diseases, such as chronic kidney disease (Hocher and Adamski, 2017), coronary heart disease (Yao *et al*., 2017) or Parkinson's disease progression (LeWitt *et al*., 2017).

In addition to an untargeted approach, targeted metabolomics is often used to identify and/or quantify a certain metabolite of interest. Using targeted metabolomic approaches to determine metabolism intermediates, e.g. acetate, ethanol, butyrate, lactate, succinate or amino acids, are usually applied to validate metabolic model prediction for single organisms (Shoaie *et al*., 2015), as well as for the development of novel constraint‐based metabolic methods (Bordbar *et al*., 2017). However, in the context of microbial communities, complex microbe–microbe or microbe–host interactions can make the task of tracking the origin of a certain metabolite almost impossible, requiring pulse–chase labelling experiments. Thus, even when the metabolite can be identified, assigning this metabolite to a single organism (microbe or host) can be challenging. The combination of metabolomics with metagenomics and metatranscriptomics can contribute towards a comprehensive understanding of metabolomics data and guide the annotation process.

## Community systems biology

The previous sections exemplified current approaches to elucidate microbial diversity, community composition, functionality and activity; all focused on unravelling the interactions that shape the microbiome. Despite all the recent advances in omics technologies, no single approach can paint a comprehensive picture of the complex interaction network in a microbial community. Therefore, interdisciplinary, multi‐omic tools and analyses are necessary for elucidating the role of the different community members and to unravel mechanisms and interactions that drive community composition and dynamics. As it is increasingly easier and more cost‐effective to generate large, multi‐omic data sets, data integration and analysis have become a time‐consuming and often challenging part in the investigation of microbial communities (Palsson and Zengler, 2010). Although still in its infancy, systems biology approaches applied to communities have attracted more and more attention over the last years (Levy and Borenstein, 2013). At the same time as systems biology approaches for single bacterial and eukaryotic microorganisms have matured, e.g. through the reconstruction of comprehensive predictive models (Islam *et al*., 2014; Bosi *et al*., 2016; Kerkhoven *et al*., 2016; Levering *et al*., 2016; Zuñiga *et al*., 2016; Bartell *et al*., 2017), a Community Systems Biology (CoSy Biology) approach has emerged (Zengler and Palsson, 2012). CoSy Biology builds on the success of systems biology approaches for single organisms. For example, multi‐omic data, as described above, can be used in the context of computational models to gain insight into community interactions (Nagarajan *et al*., 2013; Embree *et al*., 2015). It should be noted that these models generally do not consider biological noise (i.e. variation of individual cells and their growth stage) but rather assume unified growth phenotypes of members within a community. In the following sections, we will describe current efforts in the construction of mathematical models for the systematic understanding of communities, and the development of robust community models guided by multi‐omics data. In analogy to systems biology approaches for single organisms, we propose an iterative design–build–test–learn cycle (Nielsen and Olson, 2002; Paddon and Keasling, 2014) that would aid the systematic understanding of communities by combining experimental and *in silico* tools in an iterative fashion (Fig. 4).

## Kinetic modelling

High‐throughput community studies, either nucleic acid‐based, metabolome‐based or phenotypical‐based, greatly outnumber model‐based analyses (Röling *et al*., 2010). The absence of robust mathematical tools to systematically contextualize experimental information has limited our understanding of community interactions (Gonzalez *et al*., 2011). However, significant progress has recently been made towards predicting various features of communities, such as community interactions and potential spatial organization, in addition to more traditional abundance predictions. Here, we recapitulate previous efforts to understand and elucidate the function of communities using mathematical models.

Since the introduction of the concept of biological mutualism by Van Beneden one and a half centuries ago (Beneden, 1876) (Fig. 1), different models emerged in the 1920s to quantitatively describe community interactions (Lotka, 1925; Volterra, 1926). The most extensively used approach has been kinetic modelling, which provides a dynamic picture of how the components of a studied system interact and respond to certain stimuli over time (Dunn *et al*., 2003; Xu *et al*., 2011). Kinetic models can be classified as unstructured or structured and stochastic or deterministic depending on their scope, the parameters considered and the way in which they are solved. To date, structured and unstructured kinetic models are the primary choice to simulate inhibition processes within communities (Tabiś *et al*., 2014). To create structured or unstructured models, a well‐characterized community is needed in order to proceed with model computational analysis, in which often the favoured interaction modelled is mutualism. The characterization involves accounting for a number of growth‐related parameters such as experimentally determined yields, substrate uptake rates, and biomass, substrate and/or product concentrations, as well as reaction saturation and inhibition constants. Mathematically, these models involve solving a set of ordinary differential equations or partial differential equations, which represent the conservation of mass in internal and external cell processes like mass conversion or exchange. The experimental and estimated parameters included into the model will depend on the community complexity. When experimental measurements of all parameters are not available, which is often the case for single microorganisms and complex communities, approximation of parameters becomes time‐consuming and computationally intensive (Bordbar *et al*., 2014).

### Unstructured kinetic models

Unstructured models simulate dynamics of biomass production by treating each individual organism as a ‘black box’. These models are mainly based on Monod‐type relationships. Unstructured kinetic models vary in complexity, from the simplest representation of a population's dynamic behaviour (basic growth principles; Huston and Smith, 1987; Dunn *et al*., 2003) to models involving vast amount of parameters related to environmental conditions, fitness, sensing and adaptation (Cabello *et al*., 2014). The most complex models describe the interplay between physical and chemical properties of the studied condition, as well as participant‐specific biological parameters such as rates of maintenance, inhibition, biomass formation or cell death (Alhumazi and Ajbar, 2005; Wade *et al*., 2016). A number of unstructured growth models for communities have been produced over the decades (Faust and Raes, 2012), the majority of which were applied to microbial ecology questions with emphasis on mutualistic systems (Wade *et al*., 2016).

Unstructured models can be used to simulate various community interaction types such as inhibitory behaviours or cross‐feeding. In 2016, Germerodt *et al*. (2016) studied the growth of six different *Escherichia coli* strains assuming cross‐feeding, where each strain differed in its requirement to obtain certain amino acids. The Cellular Automaton of Bacterial Cross‐feeding (CELL‐ABC) tool was used for simulation purposes, using as input parameters growth rates, amino acid concentrations and amino acid diffusion rates. Over time, the different genotypes in the community became stable. The simulations allowed predicting the performance of species over time and the codependency between them (Germerodt *et al*., 2016).

Furthermore, community models have also been created based on reconstructed devices that represent artificial organ systems, for example the ‘minigut’, which is a device that simulates the peristalsis of the human colon under controlled conditions (Cremer *et al*., 2016). The minigut was deployed to study a commensal synthetic community of two *E. coli* strains, in which one strain was engineered to produce galactose and the other to consume this sugar (Fig. 4). Simulations predicted strain‐specific abundances related to the position in the device over time, suggesting that flow rate and mixing play a critical role in shaping the microbiota of the colon.

Another intriguing example for unstructured model is the recently published model for a co‐culture consisting of *Monoraphidium* and *Chlamydomonas* species that predicts the coexistence or exclusion (by abundance) of each community member. This work links the modern coexistence theory and the contemporary niche theory (Letten *et al*., 2017). The modern coexistence theory is based on the overlap of two mechanisms mediating the coexistence. The first is equalization in which the fitness between active species is considered, and the second is stabilization, which encompasses the environmental conditions surrounding the community. On the other hand, the contemporary niche theory considers two species competing for limited resources. The model considers empirical relationships between supply ratio, impact niche (weight of certain member on the environment) and requirement niche (nutrients demand) resulting in the prediction of the degree of stabilization and equalization of the community.

### Structured kinetic models

The goal of structured models is to predict population behaviours based on each organism's capabilities. In structured modelling approaches, the biological system is broken down into specific compartments for each member of the population. Parameterization of these models may include such properties as the increase of an accumulated polymer (polyhydroxyalkanoates, lipids, etc.; Meeuwse *et al*., 2012; Volova *et al*., 2014), the mass transportation in and out of organelles (Cortassa *et al*., 2009), the synthesis of outer membrane vesicles (Berleman and Auer, 2013) or the metabolite exchange amongst the community through the shared space (e.g. culture medium; Wang and Papenguth, 2001; Mee *et al*., 2014). Modelling approaches follow basic principles such as mass and energy conservation (Resat *et al*., 2011). In addition, structured models provide information about specific cellular components, such as metabolites, cellular structure and its organization. Examples of structured models include a model to simulate plasmid stability in a community containing wild type and recombinant microorganisms (Zhang *et al*., 1997; Dunn *et al*., 2003).

One of the primary challenges in kinetic modelling (for unstructured as well as structured kinetic models) of monocultures and microbial communities is the feasibility and accuracy of the reaction rate constant measurements such as Michaelis–Menten constant (Km), equilibrium constant (Keq) and turnover rate (Kcat) necessary to build and parameterize these models. Another challenge is the generalization of a workflow for parameter estimation (e.g. selection of automatic step size, or methods). Efforts are underway to address this limitation using machine learning‐based methods to determine kinetic parameters (McGibbon and Pande, 2013; Du *et al*., 2016), to elucidate chemical reactions (Kayala *et al*., 2011) or to characterize complex reaction mechanisms (Blurock, 2003). However, these methods have primarily been applied to monocultures, and at large have not been expanded to include communities yet in part due to the challenge to accurately measure required parameters in a community setting.

## Constraint‐based models

While simplified constraint‐based models exist (e.g. the *E. coli* core model; Schellenberger *et al*., 2011), the power of these models lies in the scope to represent biochemical and genetic features at genome scale. The vast majority of constraint‐based models are genome‐scale representations of the metabolism of an organism, rooted in the genome sequence of the organism and topology of its own metabolic network. There are two types of genome‐scale models. One of which are the metabolic models (M‐models) and the other one type is a more comprehensive version referred to as the ME‐models. ME‐models account for highly detailed protein synthesis and degradation considering transcription units, transcription factors, tRNAs and chaperones needed for translation and protein folding. These ME‐models can also contain ion–metal enzymatic requirements for catalysis (Lerman *et al*., 2012; O'Brien *et al*., 2014) as well as translocation (Liu *et al*., 2014). To date, ME‐models have only been reconstructed for single species such as *Thermotoga maritima* (Lerman *et al*., 2012) and *E. coli* (Liu *et al*., 2014; O'Brien *et al*., 2014).

M‐models have been built for organisms for almost two decades (Edwards and Palsson, 2000), and the first community model was reconstructed a decade ago (Stolyar *et al*., 2007). M‐models are mathematically expressed by a numerical matrix of reaction stoichiometric coefficients assigned to their respective coordinates by reaction (columns) and involved metabolites (rows). The reaction stoichiometric coefficients, along with measured metabolite uptake and secretion rates, become the system constraints. Each constraint plays a critical role during simulation and prediction of growth rate depends on the accuracy of experimental data. High accuracy is especially important for determination of uptake and secretion rates, as these parameters are critical in the modelling. While these data are relatively easy to obtain for axenic cultures, those constraints are challenging to determine for individual members within a community (see Multi‐omic approaches to study microbial communities). The lack of accurate measurements for individual members (e.g. uptake rate) is one of the greatest challenges in CoSy Biology. The lack of information can therefore require multiple assumptions to be made during the parameterization step. In the case of constraint‐based community modelling, constraints such as uptake rates and the stoichiometric coefficients in the biomass reaction are parameters that often have to be obtained from the literature. The biomass reaction contains all biomass precursors (nucleotides, proteins, lipids, carbohydrates), which means that the biomass reaction of microorganisms in pure culture is assumed to be identical to the composition of these organisms existing in communities (Nagarajan *et al*., 2013). However, it is well known that different substrates or physical factors, such as temperature, can influence cellular composition, such as lipid content. The assumption that the biomass reaction is independent from environmental parameters can thus be misleading. However, most of the time organisms cannot be obtained as axenic cultures and thus the biomass reaction has to be obtained from closely related organisms (Zengler, 2008).

The reconstruction of a highly curated model for a single organism is a time‐consuming process, while reconstruction of complex community models containing many members often relies on automatically generated models (Henry *et al*., 2010). Auto‐generated models usually contain the constraints and biomass objective function of *E. coli* independently of the metabolic features of the community member and its possible role in the community (Freilich *et al*., 2011; Levy and Borenstein, 2013). Although auto‐generated models represent a valuable resource, it is important to note that these models lack the high accuracy of manually curated models. Auto‐generated models are subject of intensive gap‐filling (i.e. adding reactions and metabolites without gene associations) generating redundant results for community models. Furthermore, gap‐filling reduces representation of auxotrophies in the model, which have been shown to be critical for community assembly and maintenance (Embree *et al*., 2015).

Once constraints are applied to the model, independent of the model reconstruction process, a convex optimization method called flux balance analysis (FBA) generates an optimal solution representing the flow of mass through the network. FBA identifies the optimal solution of a given objective function. Normally, this objective is the biomass reaction (i.e. growth) which encompasses all the metabolites necessary to produce biomass (Schellenberger *et al*., 2011). FBA is based on the assumption of a steady state and is solved using linear programming. Constraint‐based models successfully predict growth rates and gene essentiality, and can accurately represent the genotype–phenotype relationship.

As these metabolic models (M‐models) are reconstructed at genome scale, they are well suited for the integration of high‐throughput community data (e.g. metabolomics, metatranscriptomics), thus enabling organism‐specific analysis and interpretation of multi‐omic data sets. Moreover, many microbial communities maintain relationships based on the exchange of metabolites, which can be assessed directly using constraint‐based analysis, as these exchange fluxes are explicitly modelled under this framework. However, it is important to note that communities are assembled and maintained not only on metabolic exchanges alone and interactions can be manifold and intertwined depending on environmental conditions (Embree *et al*., 2014; Cremer *et al*., 2016; Hibberd *et al*., 2017).

The COBRA (Constraint‐Based Reconstruction and Analysis) toolbox (Schellenberger *et al*., 2011) has been widely used to generate and solve single species and community constraint‐based models (Bordbar *et al*., 2010; Nagarajan *et al*., 2013; Hamilton *et al*., 2015). This tool allows the optimization of multiple objective functions at the same time, which is critical for communities that may be adapting to maximize the growth of multiple community members simultaneously. For example, direct interspecies electron transfer between two *Geobacter* species was simulated by optimizing the growth rate of both community members (Nagarajan *et al*., 2013). In another example of multiple objectives, the main drivers in the interaction between the bacterium *Syntrophobacter fumaroxidans* and the methanogenic archaeon *Methanospirillum hungatei* were identified and attributed to the concurring exchange of H_2_ and formate (Hamilton *et al*., 2015). Embree *et al*. (2015) used individual models to quantify the simultaneous exchange of several metabolites between the five most abundant members in a complex methanogenic community and revealed the existence of an intertwined network of amino acid auxotrophies.

M‐models can be used to predict potential competitive or cooperative interactions in microbial communities. Freilich *et al*. used large sets of auto‐generated M‐models and compared the probability of interaction between microorganisms from different ecosystems. This approach was based on similar principles of modern coexistence theory and the contemporary niche theory. The goal of the study was to use the available genome and metabolic information annotated for each identified bacteria to make predictions of community behaviour in lieu of determining strain‐specific kinetic parameters (Freilich *et al*., 2011).

Community M‐models have also been reconstructed to gain insight into human host–pathogen interaction and to determine the effect the microbiome has as an important component of human health. The first community M‐model describing host–pathogen interaction was the model of a human macrophage and the bacterium *Mycobacterium tuberculosis*. The model was able to represent three distinct pathological states and gave insights into the infection development (Bordbar *et al*., 2014).

Other modelling efforts around communities for human health applications have been focused on the human gut and bacterial interactions. The first such work focused on representative strains from the human gut, such as *Bacteroides thetaiotaomicron*,* Eubacterium rectale* and *Methanobrevibacter smithii* representing the phyla Bacteroidetes, Firmicutes and Euryarchaeota respectively. The resulting simulations of population densities amongst the species partially resemble the densities during colonization of each microorganism into germ‐free mice in different ecosystems (Shoaie *et al*., 2013). In a different approach, coexistence of community members was estimated by integrating metagenomic data with auto‐generated reconstructions of 154 models of human gut species. The authors found that control over the health of the host does not change the observed microbial patterns, indicating that species interactions are not fully defined by the host (Levy and Borenstein, 2013). One recent achievement was the reconstruction of preliminary models for 773 human gut bacteria (Magnúsdóttir *et al*., 2016). The authors used the AGORA (Assembly of Gut Organisms through Reconstruction and Analysis) toolbox to semi‐automatically create 773 genome‐scale metabolic models of the most representative microorganisms in the gut microbiome (see Fig. 1). These models are a resource and are also expected to facilitate the study of host–microbiome interactions as their nomenclature and structure are compatible with a reconstruction of human metabolism, Recon2 (Swainston *et al*., 2016). While Recon2 is not a tissue‐specific model and represents all human metabolism together, tissue‐specific models have been reconstructed in the past and can be utilized for studying specific interactions of microbial communities in the human body (Ryu *et al*., 2015; Schultz and Qutub, 2016).

Recently, various tools to increase the scope of community M‐models have been developed, see for example a recent review by Biggs *et al*. (2015). While FBA‐based modelling approaches assume a steady state, most communities are highly dynamic. To account for this, a dynamic flux balance analysis (dFBA) has been developed and extended to microbial communities to predict time‐varying interactions between species and their effects on microbial composition. This approach is referred to as dynamic multispecies metabolic modelling (Zhuang *et al*., 2011). The CASINO toolbox (Community And Systems‐level INteractive Optimization) was created as a comprehensive platform to analyse gut microbial communities (Shoaie *et al*., 2015). The authors used experimental data from 45 obese and overweight individuals to validate their predictions. The predictions quantitatively describe exchange of metabolites in response to diet intervention (Shoaie *et al*., 2015).

The success of genome‐scale models in predicting community interactions has been empowered by the high quality of single organism reconstructions used to generate community M‐models. Presently, a challenge in constraint‐based community modelling is the reconstruction of reliable models from either single‐cell sequencing or from genomes obtained by differential binning of metagenomics data (Martinez‐Garcia *et al*., 2012). A current limitation of this approach is that microorganisms described solely by omics tools are typically poorly characterized, and thus modelling approaches rely on genome information only. The continuous contextualization of omic data will help to improve these models so that they can accurately predict how a microorganism or a metabolite benefits human health (see Fig. 4).

A hybrid modelling concept that combines kinetic and constraint‐based modelling is cybernetic modelling (Dhurjati *et al*., 1985). Here, the models account for metabolic regulation as in kinetic models, while considering a cellular objective function similar to constraint‐based modelling. Cybernetic modelling has been extended to microbial community modelling in describing the interaction of the yeasts *S. cerevisiae*,* Pichia stipitis* and *Kluyveromyces marxianus* (Geng *et al*., 2012). Those studies focused on the inference of enzyme kinetic parameters and reaction thermodynamics for the production of bioethanol within the metabolic pathways of interest in each member. Another hybrid model is known as whole‐cell computational model (Karr *et al*., 2012), which was used to predict certain stages of interactions between the human host and *Mycoplasma genitallium*. This model considers the main processes inside a cell, such as the synthesis of protein, RNA, DNA and metabolite levels. All processes are explained in the context of cell geometry, external stimulus, time and host–pathogen interaction (Karr *et al*., 2012).

## Computational tools for data enrichment and analysis

Both constraint‐based and kinetic modelling approaches rely heavily on experimental data necessary to reconstruct and constrain these models. Advances in computational tool development have been going hand in hand with advances made in molecular biology. The latest advances in database development, network visualization, statistical approaches and other mathematical tools to produce and analyse high‐throughput community data have played an essential role in CoSy Biology. For example, large databases including the human and the earth microbiome projects (Turnbaugh *et al*., 2007; Gilbert *et al*., 2010; Methé *et al*., 2012) are now available. Furthermore, expanded metabolic databases such as KEGG (Kanehisa *et al*., 2014), MetaCyc (Caspi *et al*., 2014), GNPS (Wang *et al*., 2016) and tools for identification and annotation of metabolites using Bayesian approaches (Silva *et al*., 2014) became recently available online. In addition, new software and graphical visualization tools have been generated to ease interpretation and presentation of big data (King *et al*., 2015).

In addition to constraint‐based and kinetic modelling, statistical models have also been employed to study community behaviour. One such variation of unstructured models is the various correlation approach (e.g. Pearson's product–moment coefficient, rank correlation coefficients, sensitivity and data distribution; Bersanelli *et al*., 2016), which has been widely used for the analysis of omic experimental data. The main application of the correlation approach has been the analyses of species co‐occurrence, diversity, as well as replication rates (Roling and van Bodegom, 2014; Brown *et al*., 2016). Additionally, statistical correlations and other advanced statistical analyses (e.g. multivariable analysis) have been used to estimate community assembly, approximation of strain and expression abundance, taxa turnover and phylogenetic structure (Nemergut *et al*., 2013). One key issue with the correlation‐based approach is to establish methodologies oriented to avoid computational artefacts associated with the presence of low abundance organisms or low expressed genes within specific members of a community (Nemergut *et al*., 2013).

Correlation‐based methods require dimension reduction approaches. These approaches extract the linear relationships that best explain the correlation across omic data sets, and data variability including issues such as batch effects or outliers (Meng *et al*., 2016). One example of such a method is the QIIME toolbox for the analysis of community sequence data. This toolbox explores data integration using principal component analysis (Caporaso *et al*., 2010). A recent study used 16S rRNA gene sequence data analysed with QIIME to demonstrate the influence of pathogens on the microbiota of an arthropod (Abraham *et al*., 2017). Arthropods are agents, which trigger human infections, e.g. granulocytic anaplasmosis. The study showed that external pathogens became active and manipulated the local environment (in this case, the microbiota of the arthropod) to facilitate pathogen infection.

## Potential biotechnological applications

Insights into microbial communities are poised to change various aspects of our daily life, including human health care, animal husbandry, agriculture and fermented food industry (Gilbert *et al*., 2010; Sekirov *et al*., 2010; Fouts *et al*., 2012; Ivey *et al*., 2013; Shoaie *et al*., 2013; McDonald *et al*., 2016; Knight *et al*., 2017). Multi‐omic data generation and subsequent computational modelling are key to our understanding of the microbiome. The hope is that this knowledge will enable direct or indirect modulation of the microbiome (e.g. in the form of pro‐, post‐ or prebiotics) to benefit human life, improve biotechnological application or promote a healthier environment. Model‐driven data analysis has already associated host–microbiome features to human wellness and health and has even been deployed for forensic purposes (Sekirov *et al*., 2010; Shoaie *et al*., 2015; Javan *et al*., 2016a,b). Additionally, microbiomes of livestock and plants have also been exploited to promote increase in agricultural productivity (Andreote and Pereira e Silva, 2017; Thibodeau *et al*., 2017). It has thus been a long‐standing goal of the biotechnology and medical industry to understand and control the function of microbial communities. Besides bacteria and fungi, viruses represent an inherent part of the microbiome. The viral microbiome not only plays a major role in the environment (Zeigler Allen *et al*., 2017) and human health (Cadwell, 2015) but also is of critical importance for the bioprocess industry (Marco *et al*., 2012). Viral contamination of cell‐based fermentations is responsible for hundreds of millions of dollars in lost revenue per year. Computational modelling of the virome in context of the microbiome could provide new strategies to combat viral infections (Maynard *et al*., 2010). This section focuses on biotechnological applications enabled by new insights into microbial communities and the human microbiome (see Fig. 1).

## Data‐driven advances in human health

Human individuals have a unique microbiome that has a profound impact on their health. As such, personalized medicine has the potential to play an important role in guiding wellness and providing possible biotherapies targeting the microbiome (McDonald *et al*., 2016). Microbiome‐based therapies (McDonald *et al*., 2016; Nature‐Biotechnology, 2017) can be classified into the following categories: prebiotic, probiotic, post‐biotic and modulation treatments. However, identification of what constitutes a healthy microbiome, which microorganism is beneficial for a defined outcome and determining the causal effects of a manipulated microbiome remain key challenges for the field.

Prebiotics are composed primarily of non‐human digestible food or ingredients that promote the growth of beneficial microorganisms inside the host. Recent studies have shown promising results for the use of prebiotics to counter obesity in adults and improve wellness in infants (Lyon *et al*., 2011; Koleva *et al*., 2015; Nature‐Biotechnology, 2017). Probiotics consists of individual beneficial microorganisms or combinations thereof (Gibson *et al*., 2017). Probiotics can be either consumed or applied topically and have been shown to confer positive effects in a variety of areas of human health (Holz *et al*., 2017). Natural probiotics have a long history of success in human gut health and have shown promise in treating inflammatory bowel disease, necrotizing enterocolitis, allergies, urogenital problems and skin imbalances (Nakatsuji *et al*., 2017). However, administration of probiotics does not necessary re‐establish the microbiota to ‘normal’ levels (Petschow *et al*., 2013; Holz *et al*., 2017). Currently, there is still a lack of understanding about the mode of action and collateral effects of probiotic treatment on human health. Post‐biotics consist of metabolic products of non‐live probiotic microorganisms that have been shown to grant favourable effects onto the host health (Klemashevich *et al*., 2014); especially, the short‐chain fatty acids, acetate, propionate and butyrate have shown encouraging outcomes for the treatment of different diseases, such as colitis, arthritis, asthma, gout or pneumonia (Thomaz *et al*., 2016).

Finally, microbiome transplants, which can be classified as modulation methods (Olle, 2013), involve the administration of live microorganisms from a donor into a recipient in order to re‐establish the healthy microbiome of the recipient. These transplants have been attempted for a variety of different diseases. The first transplants were used to successfully treat urinary tract infections by implanting *Lactobacillus crispatus* (see Fig. 1), which promoted recolonization of the urinary tract and eventually led to the restoration of health of the recipient (Olle, 2013). Skin microbiome transplants have also shown positive effect for the treatment of atopic dermatitis (Myles *et al*., 2016; Nakatsuji *et al*., 2017). Faecal microbiome transplants have proven to be useful for the treatment of *Clostridium difficile* colitis (Gupta *et al*., 2016) and various companies are currently developing probiotics as therapy for *C. diff* colitis. Preliminary results have also suggested potential use of faecal transplants for the treatment of inflammatory bowel disease, obesity, metabolic syndrome and functional gastrointestinal disorders (Thomaz *et al*., 2016). There have been a number of attempts to define methods and apparatuses for faecal transplant, but robust and reliable methods are still under development (Gupta *et al*., 2016; Kumar *et al*., 2017). One of the main limitations of faecal microbiome transplants has been defining standards for effective microbiome profiles and appropriate donors.

There have been a number of other applications targeting the microbiome to improve human health, prolong wellness and for diagnostic medicine. Two recent microbiome studies have made progress towards drafting a generalized human diet in the context of ageing and human longevity (Heintz and Mair, 2014; Shoaie *et al*., 2015). Additionally, multi‐omic tools applied to the human microbiome have shown promise towards the discovery of biomarkers, such as saliva‐based biomarkers useful for the detection of oral cancer, Crohn's disease, pancreatic cancer, chronic pancreatitis, periodontal disease, dental caries and obesity (Yoshizawa *et al*., 2013).

## Microbiomes in forensic analysis

Multi‐omics data of deceased subjects have resulted in defining the thanatomicrobiome (Javan *et al*., 2016a), in which insights into post‐mortem microbial colonization (putrefaction) of external and internal parts of the human body establish the basis for forensic applications. Recently, studies have proposed that multi‐omic microbiome tools may be a valuable resource to generate markers for deciphering human ante‐mortem and post‐mortem events (Javan *et al*., 2016a,b). A longitudinal study performed on 12 female and 15 male cadavers demonstrated significant differences in microbiome colonization by organ (brain, buccal cavity, heart, liver and spleen) over the course of 240 h after death. The determination of microbiome profiles in each organ as putrefaction progresses lays the foundations for estimating post‐mortem interval times (Javan *et al*., 2016b). In addition, amplicon sequencing, shotgun metagenomics and metabolomics were proposed as tools for the contextualization of ante‐mortem evidence (Metcalf *et al*., 2017). The hope is that the origin of soil samples collected from cadavers could be geographically located using for example the Earth Microbiome Project database (Gilbert *et al*., 2010). Also, the skin microbiome is highly diverse and can be perturbed by environmental conditions or by being in contact with objects before and after death. It has been hypothesized that skin microbiome features can provide clues of cohabitation patterns, lifestyle habits, medication and daily ante‐mortem routines of the subject (Metcalf *et al*., 2017). Standardized methods and protocols of the thanatomicrobiome are currently under development, and these multi‐omic data studies could become a reliable tool in forensic analysis.

## Microbiome methods for agriculture and livestock production

The microbiomes of plants and animals have recently become a primary interest for the agricultural industry to improve production. The microbiome of the rhizosphere, which is the narrow region of soil that is directly influenced by root secretions and the associated microorganisms, as well as different livestock animals, have been studied to determine inroads for improvement (Brulc *et al*., 2009; Berendsen *et al*., 2012; Isaacson and Kim, 2012). Plant‐associated microbiomes vary substantially depending on species of plant, the location on the plant, e.g. leaves, branches or roots (Wagner *et al*., 2016), as well as on the soil and its condition. Manipulating plant microbiomes has become a promising strategy for agricultural plant growth management through increasing nutrient‐use efficiency, abiotic stress tolerance and disease resistance in the short term (Gopal *et al*., 2013; Busby *et al*., 2017). Data‐driven analysis has been focused on empowering the growth of plants as an alternative to reduce the use of synthetic fertilizers and pesticides. Increased production yields have been obtained by coating seeds with beneficial microbes (O'Callaghan, 2016). In addition, plant‐probiotics supplementation into the soil has been proven to enhance the production of crops like soybean, wheat and corn (Rascovan *et al*., 2016). Uncovering the nature and significance of microbial–plant interactions remains a serious effort for the field, and currently, plant–microbiome experimental studies greatly outnumber modelling studies of plants, due to the inherent complexity of plant genomes and the resulting reconstruction efforts. However, model‐driven multi‐omic analysis of plant microbiomes has the potential to improve agricultural productivity, especially in the face of human population growth and climate change (Andreote and Pereira e Silva, 2017).

Analogous to plant improvement efforts, the demand for efficiency continues to increase also for the livestock sector (Council, 2015). While health is a determining factor for livestock as well, efforts for improvement are targeting performance (e.g. weight and growth) as well as cost/benefit. Modelling and data‐driven tools applied to livestock will likely contribute to the understanding and control of breeding, nutrition and animal health, in order to reach sufficient and improved, as well as biosustainable production. Multi‐omic data analysis has provided insights into beneficial effects of probiotics on the chicken gut microbiome. The study showed that chickens fed with a selenium‐yeast probiotic were more resistant to the colonization of the pathogen *Campylobacter jejuni* (Thibodeau *et al*., 2017). Understanding the role of the microbiome in livestock remains a growing area of research.

## Future directions and conclusions

The integration of experimental and computational approaches is a key driver in obtaining in‐depth knowledge of microbial communities and learning about assembly, and maintenance of communities in order to predict how microbomes will react to specific perturbations. Generation of longitudinal data has shown to be highly informative to unravel community dynamics. Incorporation of large metadata will further improve our knowledge about what members are playing what roles in complex associations. Moving forward, it is necessary to update and advance algorithm and software development in order to balance experimental data production with fast and comprehensive data analysis tools. These improvements require interdisciplinary research efforts encompassing different disciplines, such as microbiology, medicine, biotechnology, statistics, computer science and systems biology (Bersanelli *et al*., 2016; Meng *et al*., 2016).

The construction of models to characterize communities in response to stimuli has been initiated and has already yielded new insight into microbial interactions. Various models have been deployed to understand and manipulate microbial communities. In order to improve the accuracy of predictive community modelling, methods need to be developed to better parameterize kinetic models, which are often hampered by a lack of accurate measurements necessary to describe the entangled nonlinear biological processes. In the case of constraint‐based modelling, it is important to improve on automated reconstruction methods, as well as to conduct well‐designed experiments to determine exchange capabilities, as well as parameterize and validate these models. The experimental design should account for controls capturing as many details as possible, e.g. measurements of free compounds in the culture medium over time and thus delineate their origin (i.e. by secretion or lysis). Even though the genome provides evidence about transport capabilities, physiological and omic data can help to reveal activity of these transporters.

Furthermore, greater strides need to be made in determining and predicting the effect of spatial organization of cells within the community, effect of temperature, pH, osmolarity and ionic strength. The influence of these parameters is often not fully understood even for pure cultures, and as a result, their effects are rarely included in community models.

Future analysis of host–microbe interactions should be based on robust and reliable mathematical tools that can accurately elucidate different types of interactions and their underlying mechanisms. The first steps have been taken by adapting Koch's postulates using the latest experimental techniques to guide and evaluate the cause of multimicrobe diseases (see Byrd and Segre, 2016 and highlighted work therein). It is also possible to better predict drug side effects if host and microbiome are considered as actively interacting entities. Another application where knowledge about microbial interactions becomes crucial is the rational design of synthetic communities to be used in medicine and agriculture. Advances in the integration of multi‐omics data to mathematical modelling will provide unprecedented insight into microbial communities and will further advance tools on how to manipulate them.

## Conflict of interest

The authors declare no conflict of interests.
